# Indirect Modulation by FLP-1 Peptides on Chemotaxis and Dispersal Behavior in *C. elegans*

**DOI:** 10.17912/micropub.biology.000930

**Published:** 2023-09-08

**Authors:** Michael J Lynch, Alessandro S Mercado, Chris Li

**Affiliations:** 1 Biology, City College of New York, CUNY; 2 Biology, The Graduate Center, CUNY, New York, New York, United States

## Abstract

Parasitic nematodes infect and cause morbidity in over one billion people worldwide, with current anthelmintic drugs decreasing in efficacy. To date, nematodes produce more types of neuropeptides than any other animal. We are interested in the role of neuropeptide signaling systems as a possible target for new anthelmintic drugs. Although FMRFamide-related peptides are found throughout the animal kingdom, the number of these peptides in nematodes greatly exceeds that of any other phylum. We are using *Caenorhabditis elegans* as a model for examining FMRFamide-like peptides, all of which share a C-terminal Arg-Phe-amide and which are known as FLPs in nematodes. Our previous work indicated interactions between the *daf-10* , *tax-4* , and *flp-1* signaling pathways. In this paper, we further explore these interactions with chemotaxis and dispersal assays.

**Figure 1. flp-1 signaling is not required for chemosensation, but its signaling can affect the chemosensory and dispersal responses:**
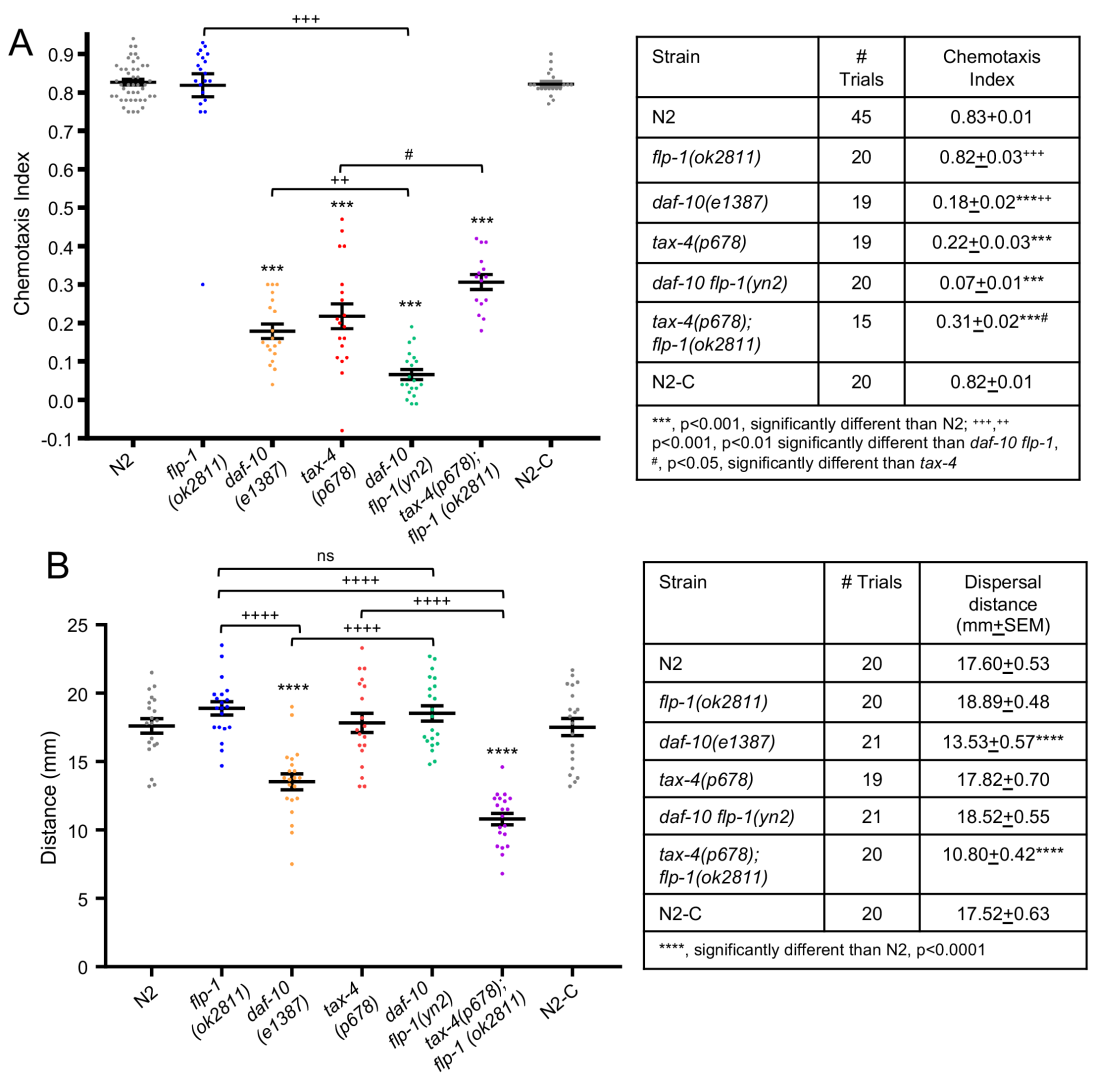
Similar to wild type, *flp-1* mutants chemotax towards benzaldehyde (A) and disperse in the absence of food (B). However, loss of *flp-1* enhances or slightly suppresses the chemosensory defects of *daf-10* and *tax-4* mutants, respectively (A)(***, p<0.001, significantly different than N2 ; ^+++^ , ^++^ p<0.001, p<0.01 significantly different than *daf-10 flp-1* , ^#^ , p<0.05, significantly different than *tax-4 )* . By contrast, loss of *flp-1* suppresses or causes dispersal defects in *daf-10* or *tax-4* mutants, respectively (B) (****, p<0.0001, significantly different than N2 , ++++, p<0.0001). Methods: Chemotaxis assays were performed and the chemotaxis index was calculated as described (Bargmann & Horvitz, 1991); each trial had at least 60 animals. For the dispersal assay, L4 animals were picked for use the following day. Six 1-day adults were transferred to a plate without food before transferring to a new plate without food; the locations of the six animals after 15 min were averaged for each plate and constituted one trial. The phenotypes were unknown to the scorers on the chemotaxis and dispersal assays. An unknown, which corresponded to N2 (N2-C), was included in all assays.

## Description

An estimated 1.5 billion people, comprising 24% of the world’s population, are infected with soil-transmitted helminths (World Health Organization; Gang & Hallem, 2016). Parasitic nematodes also adversely affect livestock (Whittaker et al., 2017; Strydom et al., 2023) and crops (Sikora et al., 2023) . Of the existing eight classes of anthelmintic therapies, resistance to three classes of anthelmintics have cropped up in livestock (Shalaby, 2013; Abongwa et al., 2017) , suggesting that resistance in humans will soon follow, such as has been found with ivermectin (Weeks et al., 2018; Hahnel et al., 2020; Panda et al., 2022) . These twin challenges, anthelmintic resistance and changing disease patterns, strongly require the need for new anthelmintic therapies.

One possible target for new anthelmintic therapies are neuropeptides and their signaling pathways. Neuropeptides are neuromodulators that influence the strength of synaptic activity. They are derived from large precursor molecules, which undergo post-translational cleavage and modifications in dense core vesicles to form active peptides, whose release can occur at synaptic and extra-synaptic sites. Nematodes contain a significantly larger number of neuropeptides than mammals (Li & Kim, 2014) . The significance of this diverse variety of nematode neuropeptides is still unclear; however, this diversity of neuropeptides provides the animals with a rich peptide toolbox to affect synaptic activity and, ultimately, behavior. Roughly one quarter of all nematodes are parasitic nematodes, an equal number of which infect animals or plants (Al-Banna & Gardner, 2022) . Understanding the functions of these nematode neuropeptides may provide insights into understanding the behavior of parasitic nematodes and into their control.

Although the synaptic connectivity of all neurons within the nematode *Caenorhabditis elegans* has been determined (White et al., 1986) , this information only includes direct synaptic connections and gap junctions. The complete connectome, one which also includes extra-synaptic connections, is slowly being elucidated. The task, however, is complicated by the modulatory and overlapping functions of neuropeptides. For instance, a large family of FMRFamide-like peptides, all of which share a C-terminal Arg-Phe-amide, is present in *C. elegans* ; these peptides are collectively referred to as the FLPs. Each *flp* gene encodes a unique set of peptide(s). The *flp-1* gene encodes multiple peptides that share a C-terminal FLRFamide (Rosoff et al, 1992) . The gene is alternatively spliced and expressed in few neurons (Rosoff et al., 1992; Nelson et al., 1998) . Loss of different FLP-1 peptides results in several behavioral phenotypes, such as defects in locomotion, nose touch sensitivity, and egg laying (Nelson et al., 1998; Waggoner et al., 1998; Buntschuh et al., 2018) .

The double mutant of *daf-10 flp-1 ( yn2 )* shows an extreme wandering behavior, which is a synthetic phenotype due to the loss of the two genes; loss of either *flp-1* or *daf-10* alone does not cause this wandering phenotype (Nelson et al., 1998; Buntschuh et al., 2018) . The *flp-1* gene lies within the first intron of *daf-10* ; the *yn2* deletion removes sequences between introns 1 and 2 of *daf-10* and exons 1-3 and part of exon 4 of *flp-1* (Buntschuh et al., 2018) . *daf-10* encodes a component of the intraflagellar transport complex A, which is necessary for sensory reception of ciliated sensory neurons (Bell et al., 2006) . Loss of both *flp-1* and *tax-4* , which encodes a subunit of a cyclic nucleotide-gated channel homologous to the vertebrate rod photoreceptor cGMP-gated channel (Komatsu et al., 1996) , also caused a wandering phenotype, although not as severe as that of the *daf-10 flp-1* double mutant (Buntschuh et al., 2018) . We wondered whether the synthetic wandering defect occurred because *flp-1* mutants have a chemosensory defect, which enhances the chemosensory defect of *daf-10* and *tax-4* mutants, thereby causing them to wander.

To assess chemosensation in the different strains, we performed assays with the chemoattractant benzaldehyde (Bargmann et al., 1993) . As previously reported, *daf-10* and *tax-4* mutants showed severe, but not total loss of chemotaxis, because of a lack of sensory perception via the ciliated neurons in *daf-10* mutants or loss of downstream receptor signaling in response to the benzaldehyde odorant in *tax-4* mutants ( Fig. 1A ) (Albert et al., 1981; Komatsu et al., 1996; Bell et al., 2006) . Residual chemotaxis response in *daf-10* and *tax-4* mutants suggests that a secondary pathway allows for a minimal benzaldehyde response ( Fig. 1A ). *flp-1* mutants showed no chemotaxis defect to benzaldehyde and performed comparably to wild-type animals ( Fig. 1A ). Hence, we expected that the double mutants, *daf-10 flp-1* and *tax-4 ; flp-1* , would show decreased chemotaxis responses similar to the *daf-10* and *tax-4* single mutants. Instead, we found that the *daf-10 flp-1* double mutant showed more severe chemotaxis defects than *daf-10* mutants alone ( Fig. 1A ), suggesting that activity from a FLP-1 circuit can affect the chemotaxis circuit. By contrast, the *tax-4 ; flp-1* double mutants showed slightly better chemotaxis relative to *tax-4* mutants alone ( Fig. 1A ), suggesting that an alternative pathway that is inhibited by the FLP-1 circuit is employed.

In the absence of food, *C. elegans* undergoes two successive behaviors: if the period of starvation is short (e.g., less than 10 minutes), animals will show localized search behavior for food; after 10 minutes, animals begin to show dispersal behavior, whereby animals make less turns, allowing them to move forward for longer runs (Gray et al., 2005) . Inactivation of the AVK interneurons, which release FLP-1 peptides, results in localized searching behavior, suggesting that tonic release of FLP-1 peptides from the AVK neurons is necessary for dispersal behavior (Oranth et al., 2018) . We examined dispersal behavior in the different strains to determine whether this could explain the wandering phenotype. Using a modified dispersal assay, we found that *flp-1* mutants dispersed in search of food similar to wild-type animals ( Fig. 1B ). By contrast, *daf-10* mutants had a significantly reduced dispersal compared to wild type ( Fig. 1B ). This decreased dispersal was suppressed in a *flp-1* mutant background ( Fig. 1B ), suggesting that a FLP-1 signaling circuit over-rides the sluggish sensory response of *daf-10* mutants. Surprisingly, although *flp-1* and *tax-4* mutants had similar dispersal rates as wild type ( Fig. 1B ) (Oranth et al., 2018) , the double *tax-4 ; flp-1* mutant shows a severely compromised dispersal defect ( Fig. 1B ).

**Discussion**

*flp-1* encodes multiple peptides of the FLRFamide family and is the only *flp* gene that has been found in all parasitic and non-parasitic nematodes to date (Li & Ki, 2014) . Loss of *flp-1* causes several defects, including locomotory and reproductive defects (Nelson et al., 1999; Buntschuh et al., 2018) . In particular, *flp-1* mutants are hyperactive with an exaggerated waveform, suggesting that the normal function of *flp-1* signaling is to inhibit locomotory and waveform circuits, which are the output of integrating multiple environmental cues. Loss of *flp-1* and *daf-10* or *tax-4* leads to a synthetic wandering phenotype, which we suggested may be due to loss of chemosensory responses in *flp-1* mutants. While loss of *daf-10* or *tax-4* causes chemotaxis defects, loss of *flp-1* had no effect on the chemotaxis response. By contrast, loss of *flp-1* in *daf-10* and *tax-4* mutants enhanced or partially suppressed the chemotaxis effects, respectively. *daf-10* is expressed in all amphidial, phasmid, cephalic, labial, mechanosensory, and BAG neurons (Perkins et al., 1986; Starich et al., 1995) , whereas *tax-4* is expressed in a subset of the amphidial neurons as well as the BAG, AUA, and URX neurons (Komatsu et al., 1996) . Hence, chemosensation of other chemicals and osmolarity responses are present in *tax-4* mutants, but not in *daf-10* mutants. We suggest that when *flp-1* is knocked out in an animal with severe sensory defects, such as *daf-10* mutants, these double mutants are hyperactive and will travel in a random, non-directed migration pattern, resulting in a low chemotaxis index ( Fig. 1A ). In *tax-4* mutants, however, some sensory responses are still present, driving a slightly larger number of *tax-4 ; flp-1* double mutants to the odorant than *tax-4* mutants alone.

In the absence of food for extended periods (e.g., over 10 minutes), wild-type animals switch from localized search forays for food to longer runs with less turns, a behavior called dispersal (Gray et al. 2005) . *daf-10* mutants did not disperse in the absence of food ( Fig. 1B ), perhaps because of its compromised sensory response. However, *tax-4* mutants also have a compromised sensory response, yet they dispersed similar distances as wild type ( Fig. 1B ), as other researchers have reported (Oranth et al., 2018) . Although *flp-1* mutants are hyperactive, they did not disperse significantly further than wild type, suggesting that speed and dispersal are unlinked. *daf-10 flp-1* mutants were able to disperse, suggesting that *daf-10* mutants are able to disperse when a *flp-1 -* mediated inhibition is lifted. We suggest that the lack of dispersal when AVK was optogenetically inhibited was not due to lack of FLP-1 peptide release, but the lack of release of a different neuropeptide expressed in AVK (Taylor et al., 2021) . Perhaps FLP-1 peptides act to inhibit release of this dispersal neuropeptide(s) or the levels of the dispersal neuropeptide(s) rise during starvation to over-ride the inhibitory activity of the FLP-1 peptides.

Although *flp-1* and *tax-4* single mutants did not have a dispersal defect, the *tax-4 ;* *flp-1* double mutants had a dispersal defect and remained in a dwelling state. Dispersal behavior is the result of several factors, including food scarcity and population density. Hence, the dispersal is an integration of many sensory cues, such as olfactory, gustatory, mechanosensory, pheromone, etc., that eventually lead to a motor response. Chemosensors, which detect immediate food deprivation, are dependent on *tax-4* signaling (Komatsu et al., 1996; Coates & deBono, 2002) , whereas prolonged starvation is more dependent on mechanosensation and other signaling pathways (Oranth et al., 2018) . Both of these pathways feed onto the AVK circuit (White et al., 1986) . We suggest that FLP-1 peptides are involved in dispersal activity through a second mechanism, perhaps through effects on PDE. We propose that during starvation, FLP-1 peptides can inhibit PDE activity so that dispersal behavior is promoted. In the *tax-4 ; flp-1* double mutants, the lack of sensory signaling and the lack of PDE inhibition decreases dispersal rates.

## Methods

**Strains.** *C. elegans* strains were grown and maintained at 20°C according to Brenner (1974). The wild-type strain used was N2 var. Bristol. Mutations used are as described in Wormbase (www.wormbase.org) and Buntschuh et al. (2018): LGIII: *tax-4 ( p678 )* ; LGIV: *flp-1 ( ok2811 ), daf-10 ( e1387 ), daf-10 flp-1 ( yn2 )* .

**Chemotaxis index (CI) assays.** Assays with a minimum of 60 worms each were conducted as described (Bargmann and Horvitz, 1991) . At least 15 trials were performed for each strain.

**Dispersal assays.** Fourth stage larval animals were picked for use the following day. 1-day adults were transferred to a plate without food; six animals were then transferred to the test plate, which had no food. The distance each animal traveled after 15 min were averaged and considered one trial. At least 19 trials were conducted for each strain.
